# Piezoelectric domain walls in van der Waals antiferroelectric CuInP_2_Se_6_

**DOI:** 10.1038/s41467-020-17137-0

**Published:** 2020-07-17

**Authors:** Andrius Dziaugys, Kyle Kelley, John A. Brehm, Lei Tao, Alexander Puretzky, Tianli Feng, Andrew O’Hara, Sabine Neumayer, Marius Chyasnavichyus, Eugene A. Eliseev, Juras Banys, Yulian Vysochanskii, Feng Ye, Bryan C. Chakoumakos, Michael A. Susner, Michael A. McGuire, Sergei V. Kalinin, Panchapakesan Ganesh, Nina Balke, Sokrates T. Pantelides, Anna N. Morozovska, Petro Maksymovych

**Affiliations:** 1 Faculty of Physics, Vilnius University, Vilnius, LT-01513 Lithuania; 2 The Center for Nanophase Materials Sciences, Oak Ridge National Laboratory, Oak Ridge, TN 37831 USA; 3 Department of Physics and Astronomy and Department of Electrical Engineering and Computer Science, Vanderbilt University, Nashville, TN 37235 USA; 4 University of Chinese Academy of Sciences & Institute of Physics, Chinese Academy of Sciences, Beijing, China; 5 Institute for Problems of Materials Science, National Academy of Sciences of Ukraine, Krjijanovskogo 3, 03142 Kyiv, Ukraine; 6 Institute of Solid State Physics and Chemistry, Uzhgorod University, 88000 Uzhgorod, Ukraine; 7 Neutron Scattering Division, Oak Ridge National Laboratory, Oak Ridge, TN USA; 8 Materials and Manufacturing Directorate, Air Force Research Laboratory, WPAFB, OH 45433 USA; 9 UES, Inc. 4401 Dayton-Xenia Rd., Dayton, OH 45432 USA; 10 Materials Science and Technology Division, Oak Ridge National Laboratory, Oak Ridge, TN 37831 USA; 11 Department of Electrical Engineering and Computer Science, Vanderbilt University, Nashville, TN 37235 USA; 12 Institute of Physics, National Academy of Sciences of Ukraine, Prospect Nauky 46, Kyiv-28, 03680 Ukraine

**Keywords:** Ferroelectrics and multiferroics, Surfaces, interfaces and thin films

## Abstract

Polar van der Waals chalcogenophosphates exhibit unique properties, such as negative electrostriction and multi-well ferrielectricity, and enable combining dielectric and 2D electronic materials. Using low temperature piezoresponse force microscopy, we revealed coexistence of piezoelectric and non-piezoelectric phases in CuInP_2_Se_6_, forming unusual domain walls with enhanced piezoelectric response. From systematic imaging experiments we have inferred the formation of a partially polarized antiferroelectric state, with inclusions of structurally distinct ferrielectric domains enclosed by the corresponding phase boundaries. The assignment is strongly supported by optical spectroscopies and density-functional-theory calculations. Enhanced piezoresponse at the ferrielectric/antiferroelectric phase boundary and the ability to manipulate this entity with electric field on the nanoscale expand the existing phenomenology of functional domain walls. At the same time, phase-coexistence in chalcogenophosphates may lead to rational strategies for incorporation of ferroic functionality into van der Waals heterostructures, with stronger resilience toward detrimental size-effects.

## Abstract (web summary)

Domain walls in van der Waals layered ferrielectric CuInP_2_Se_6_ exhibit piezoelectric response. This striking departure from traditional ferroelectric behavior is ascribed to a partially polarized antiferroelectric state, where the domain wall separates coexisting regions of ferrielectric and antiferroelectric phases.

## Introduction

Layered thiophosphates, with a general composition of CuInP_2_*Q*_6_ (*Q* = S, Se)^1^, have recently gained attention as candidate materials for two-dimensional^2–4^ (2D) or few-layered ferroelectrics^2,5^. The sulfur^6,7^ and selenium^8–10^ compounds have similar structure of individual layers and a concomitant ferrielectric (FE) ordering, with Cu^+^ and In^3+^ ions counter-displaced within individual layers, against the backbone of P_2_*Q*^*4-*^_6_ anions. The spontaneous polarization of the sulfide can range from ~5 μC/cm^2^ to ~12 μC/cm2 ^11^ vs ~2.5 μC/cm^2^ ^12^ in the selenide, in part due to larger off-centric Cu displacement in the sulfide. Despite the structural similarity, the reported properties of their phase transitions are quite different^10^. Other than the difference in the transition temperatures (~230 K^8,9^ in the selenide vs ~305 K in the sulfide^6,10^), CuInP_2_Se_6_ exhibits a broader transition window compared to CuInP_2_S_6_, as evidenced by macroscopic dielectric, caloric and thermal characterization^9,10^. It was proposed that this anomaly is evidence for the coexistence of ferrielectric and antiferroelectric (AFE) ordering, and an incommensurate phase that precedes ferroelectric ordering^9^. The properties of the intermixed S–Se compound are even more interesting, possibly involving a Lifshitz transition as well as polar glassy phases^13^. The apparent compatibility of chalcogenophosphates with a variety of polar orderings signifies comparatively weak dipolar correlations in the lattice. This property may be particularly pertinent toward prospective application of these materials as functional components of van der Waals heterostructures.

Indeed, recently, Song et al.^12^ proposed that ultrathin films of CuInP_2_Se_6_ develop an antiferroelectric ground state, with the ferrielectric-antiferroelectric crossover occurring at a thickness of ~6–8 layers. The primary driving force for the crossover is the depolarizing field that favors the antiferroelectric with net zero polarization. This feature is in contrast to perovskite oxides, such as the canonical BaTiO_3_, which become non-polar in the ultrathin limit^14^. However, at present, most nanoscale polar properties in both CuInP_2_S_6_ and CuInP_2_Se_6_ remain to be understood, with respect to the mechanisms that screen spontaneous polarization at the interfaces, polarization switching, and the structure of the domains and their domain walls^1^ as well as the scalability down to the single-layer limit. Understanding these behaviors will also help to identify the possible mechanisms by which these materials can be functional in van der Waals heterostructures.

Here, we report the structure of polarization domains in bulk CuInP_2_Se_6_ utilizing quantitative imaging of nanoscale piezoelectric properties. Contrary to the expectation of ferrielectric ordering analogous to CuInP_2_S_6_, we reveal a fundamentally different domain structure, with two markedly different values of piezoresponse. Moreover, the domain boundaries exhibit the strongest piezoelectric response, with up to fourfold enhancement compared to domain surfaces. CuInP_2_S_6_, on the other hand, features an expected domain structure, with nearly uniform value of piezoresponse within domains, alternating polarization orientation and domain walls with vanishing piezoresponse^15–17^. Despite the more complex polar structure of CuInP_2_Se_6_, the domains can be flexibly manipulated with applied fields. We explain the phenomena observed in CuInP_2_Se_6_ by considering the real-space domain structure of an antiferroelectric that is partially polarized in finite electric field. Our arguments are supported by Raman spectroscopy and DFT calculations indicating the possible coexistence of ferrielectric and antiferroelectric states. FE/AFE coexistence presents an intriguing opportunity for few-unit-cell thiophosphates, particularly within van der Waals heterostructures. At the same time, piezoelectric domain walls, which are polar phase boundaries in this case and can be readily manipulated by applied electric fields, present a new functional element for the domain-wall electronics paradigm^18,19^.

## Results and discussion

**Fig. 1:**
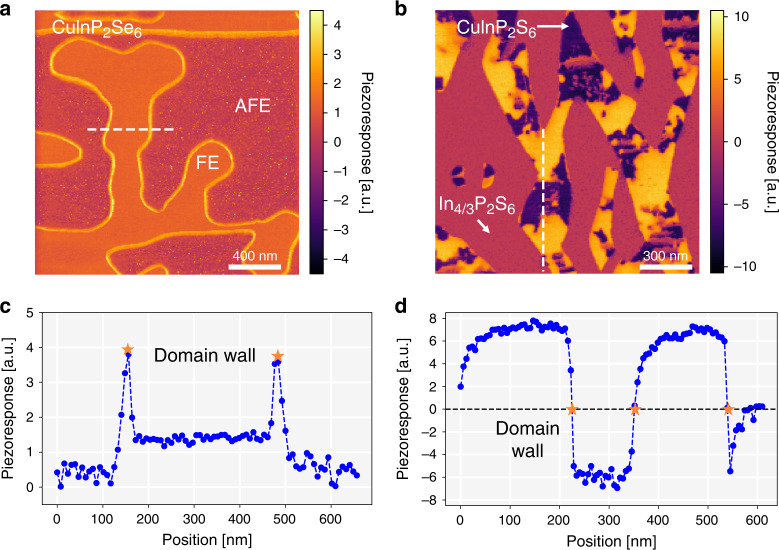
Comparison of piezoresponse due to domains and domain walls in CuInP_2_Se_6_ and Cu_0.4_In_1.2_P_2_S_6_. CuInP_2_Se_6_ surfaces were measured at 140 K in ultrahigh vacuum and that of CuInP_2_S_6_—at room temperature in controlled environment: **a**, **b** piezoresponse signals *A*cos(*θ*) (where *A* is the amplitude and θ the phase of piezoresponse)—revealing at least three distinct magnitudes of piezoresponse for both materials, and **c**, **d** line-profiles of piezoresponse along the white dashed lines, crossing domain boundaries in **a**, **b**, respectively. Orange arrows point to the regions of the domain walls.

Piezoresponse force microscopy does not exhibit any significant signal on the surface of CuInP_2_Se_6_ at room temperature in the paraelectric state, as expected. On a crystal cooled below 200 K, the piezoresponse phase and amplitude images (Fig. 1a, b) reveal domains of various forms and sizes (the measured response is defined by the normal components of the piezoelectric coefficient $d_{3j} = 2\varepsilon _0\varepsilon _{33}Q_{j3}P_3$, where the *Q*_*j*3_ are components of electrostriction tensor, *P*_3_ is the normal polarization component, and *ε*_33_ is the normal component of the dielectric constant). Despite structural similarity, however, there is a stark contrast between CuInP_2_Se_6_ and Cu_0.4_In_1.2_P_2_S_6_ as shown in Fig. 1. In Cu_0.4_In_1.2_P_2_S_6_, the contrast of the piezoelectric signal.

comprises the non-polar dielectric phase In_4/3_P_2_S_6_, with negligible piezoresponse, and the ferrielectric phase CuInP_2_S_6_, whose piezoresponse is comparable to a ferroelectric: domains of opposite polarization have opposite signs of piezoresponse, with nearly zero signal at the domain wall in between (Fig. 1b, d)^20,21^. Cancellation of piezoresponse at the domain wall is a mutual consequence of nearly zero polarization at the wall, and possibly the mechanical cancellation effect due to opposite direction of surface deformation in the adjacent domains separated by the domain wall.

**Fig. 2:**
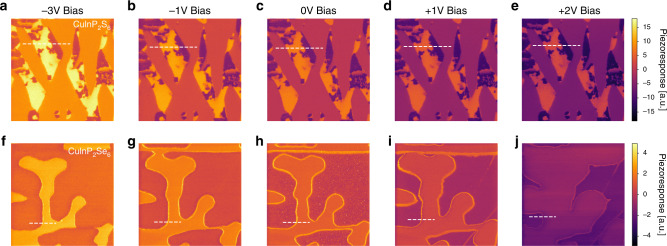
Bias-dependent piezoresponse imaging of CuInP_2_S_6_ and CuInP_2_Se_6_. **a**–**e** CuInP_2_S_6_ imaged (image size 1.5 µm x 1.5 μm) at room temperature in a controlled environment and **f**–**j** CuInP_2_Se_6_ imaged (image size 2.0 µm x 2.0 μm) at 140 K in ultrahigh vacuum. Dotted white lines indicate line profile locations for Fig. 3(a, b). The bias applied to the tip is specified in the top row.

In CuInP_2_Se_6_, extended regions of negligible piezoresponse and extended regions of uniform piezoresponse also exist. However, in contrast to Cu_0.4_In_1.2_P_2_S_6_, CuInP_2_Se_6_ crystals studied here are nearly stoichiometric, excluding the existence of a non-ferroelectric phase, such as In_4/3_P_2_S_6_ in Cu_0.4_In_1.2_P_2_S_6_. Moreover, the regions of finite piezoresponse appear only below the transition and can be flexibly manipulated with applied fields (shown below in Fig. 2j). At the same time, the piezoresponse signal across a boundary separating the two distinct regions in CuInP_2_Se_6_ has maximum rather than minimum piezoresponse signal. These observations imply that CuInP_2_Se_6_ below the ferroic transition intrinsically exhibits at least two distinct structural phases. The imaging results are reproduced for a variety of tips, cleaved surfaces and are also observed irrespective of the underlying topography (Supplementary Fig. 1). By using PFM in ultrahigh vacuum on freshly cleaved and nearly atomically flat surfaces, as well as invoking the band-excitation methodology for piezoresponse^22^, we have further ruled out simple experimental artifacts such as the underlying topography^23^, electrochemistry^24^, or mechanical properties of the contact^25^.

Further insight as well as confirmation of the above assignments is evidenced from the electric-field dependence of the measured piezoresponse (Fig. 2). It was previously pointed out that it is essential to check local electromechanical measurements for possible artefacts, such as the contribution of extrinsic electrostatics^24^. As seen in Fig. 2, the dominant effect of applied bias is to impose an offset on the measured signal in both Cu_0.4_In_1.2_P_2_S_6_ and CuInP_2_Se_6_, while keeping the contrast between entities largely unchanged.

**Fig. 3:**
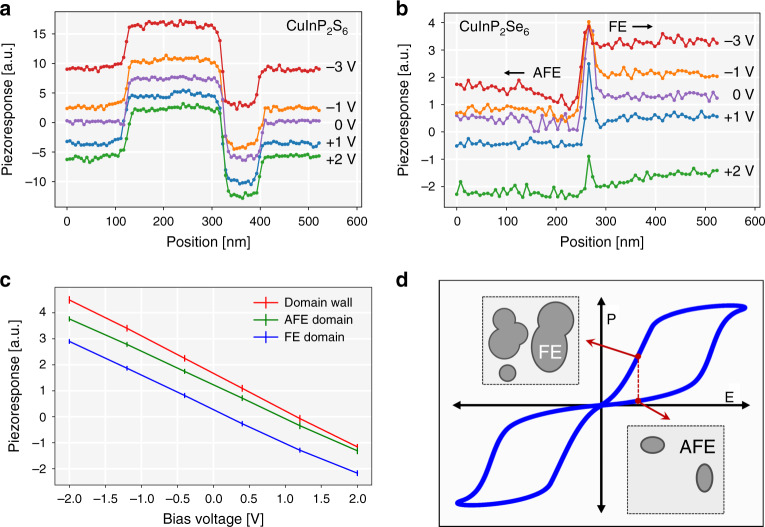
Comparison of bias-dependent piezoresponse between CuInP_2_S_6_ and CuInP_2_Se_6_. Piezoresponse line scans presented in Fig. 2(a–j) for **a** CuInP_2_S_6_ and **b** CuInP_2_Se_6_ at DC biases ranging from −3V to +2 V. **c** CuInP_2_Se_6_ piezoresponse as a function of applied voltage for domain wall, AFE domain, and FE domain derived from Supplementary Fig. 2(d) Schematic of a canonical double-hysteresis loop for antiferroelectrics. Insets illustrate a schematic top view of the surface, with spatially separated AFE (light gray) and FE (dark gray) domains.

This conclusion is further confirmed by analysis of select 1D profiles from the data in Fig. 2, as shown in Fig. 3. Indeed, relative enhancement of piezoresponse is maintained irrespective of applied field (Fig. 3a, c), while domain walls in Cu_0.4_In_1.2_P_2_S_6_ reveal approximately average signal between up and down-oriented domains (Fig. 3b). At the same time, at both −3V (Fig. 2f) and +2 V (Fig. 2j), the domain structure of CuInP_2_Se_6_ begins to evolve in applied field, respectively shrinking and growing the regions of finite piezoresponse.

Given all of the above measurements, we propose that CuInP_2_Se_6_ exhibits coexistence of antipolar and polar regions, identified, respectively, by zero and finite piezoresponse signals. The domain walls are then the boundaries separating these regions. Given the propensity of CuInP_2_Se_6_ toward antiferroelectric ordering (^12^ and see below), the polar and antipolar regions would correspond to ferrielectric and antiferroelectric phases, respectively.

This scenario of phase separation can be rationalized by considering the real-space structures underpinning the hallmark property of antiferroelectrics—a double-hysteresis switching loop (Fig. 3d)^26–28^. Double hysteresis corresponds to the switching of antiferroelectric configuration with net zero polarization to ferroelectric structure with nonzero polarization. Uniform antiferroelectric structure is, therefore, expected only in a very small range of applied fields^27^. At finite field, the system exhibits a state of finite polarization. In the real space, the structure with finite polarization can manifest as two distinct states: either the system is uniformly polarized in an applied field, or it exhibits non-uniform distribution of polarization (schematically shown in the inset of Fig. 3d), such that local regions of ferrielectric and antiferroelectric phases emerge. We believe the second case is the appropriate representation of CuInP_2_Se_6_ in our measurements.

**Fig. 4:**
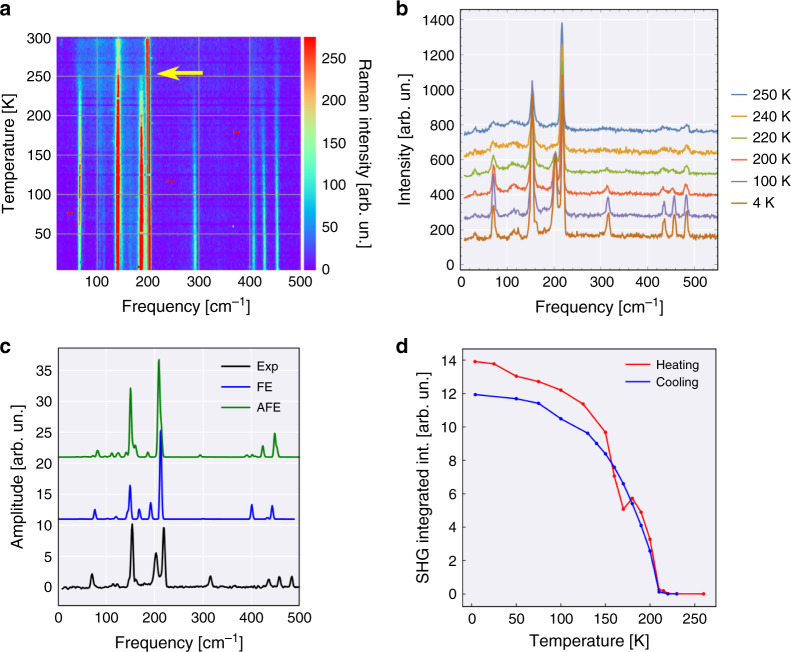
Phase transition in CuInP_2_Se_6_ detected by optical spectroscopies. **a** Temperature dependent Raman spectroscopy of a CuInP_2_Se_6_ crystal, represented as a heat map. The phase transition below ~250 K is clearly visible (yellow arrow). **b** Individual Raman spectra at several measured temperatures. **c** Comparison of the Raman spectrum at 7 K to the DFT calculations for the ferroelectric (FE) and antiferroelectric (AFE) configurations. **d** The intensity of second harmonic generation (SHG) across the phase transition at 200 K.

Our argument for the coexistence of ferrielectric and antiferroelectric phases in CuInP_2_Se_6_ is supported by Raman spectroscopy and DFT calculations. Both Raman and SHG spectroscopy, carried out on freshly cleaved crystalline flakes of CuInP_2_Se_6_, clearly detect the transition below ~250 K (Fig. 4a, d). The peaks at 203 and 219 cm^−1^ in Raman spectrum appear first at around 250 K (Fig. 4a, b) and their integrated intensity continues to grow, eventually saturating below ~180 K (Supplementary Fig. 3). Simultaneously measured SHG intensity (Fig. 4d) shows a rapid increase below 220 K. Notably, SHG detects breaking of the inversion symmetry, therefore implying the development of either ferri- or ferroelectric ordering in the probed volume of the technique.

Further, we compared the experimental Raman spectra to those calculated by DFT in the ferrielectric and antiferroelectric ground states, as shown in Fig. 4c. The AFE state in these calculations corresponds to an alternating displacement of Cu atoms along the b (or a) crystallographic direction, between opposite sides of each layer (Supplementary Fig. 4b), consistent with the prior work^12^. The In^3+^ in this case is located near the equatorial plane of each layer. In contrast, in the ferrielectric state, the Cu^1+^ and In^3+^ are offset in opposite directions relative to the midplane of the layers, but the offsets of each are identical within each cation sublattice.

The characteristic calculated signature of the AFE state appears to be a peak around 300 cm^−1^. It is negligible for the calculated spectra for the FE configurations, while prominent in the experiment (Fig. 4c). Meanwhile, the three-peak structure between 400 cm^−1^ and 500 cm^−1^ is best captured by a combination of the FE and AFE states. The probing volume of piezoresponse force microscopy measurements are comparable to the radius of the tip, ~20–50 nm, while that of optical spectroscopies ~ 1μm; thus, both are still probing near-surface regions. Note, the X-ray diffraction study of CuInP_2_Se_6_ across the phase transition, on the other hand, shows that the structure of

CuInP_2_Se_6_ belongs to the non-centrosymmetric space group *P*31*c* (No. 159) at 100 K and 180 K (Supplementary Table 1a), Supplementary Fig. 5). At 250 K, the best fit is to the centrosymmetric space group *P*$\bar 3$ 1*c* (No. 163) (Supplementary Table 1c)). These results largely agree with previous work^8^, indicating order-disorder type ferrielectric ordering in CuInP_2_Se_6_. Bulk-averaging measurements are therefore most consistent with ferrielectric ordering (Supplementary Fig. 5, Supplementary Fig. 6). However, in light of the similar energies between ferroelectric and antiferroelectric states (Supplementary Fig. 4), it is likely that uniform states of either kind can be preferred depending on specific experimental conditions.

**Fig. 5:**
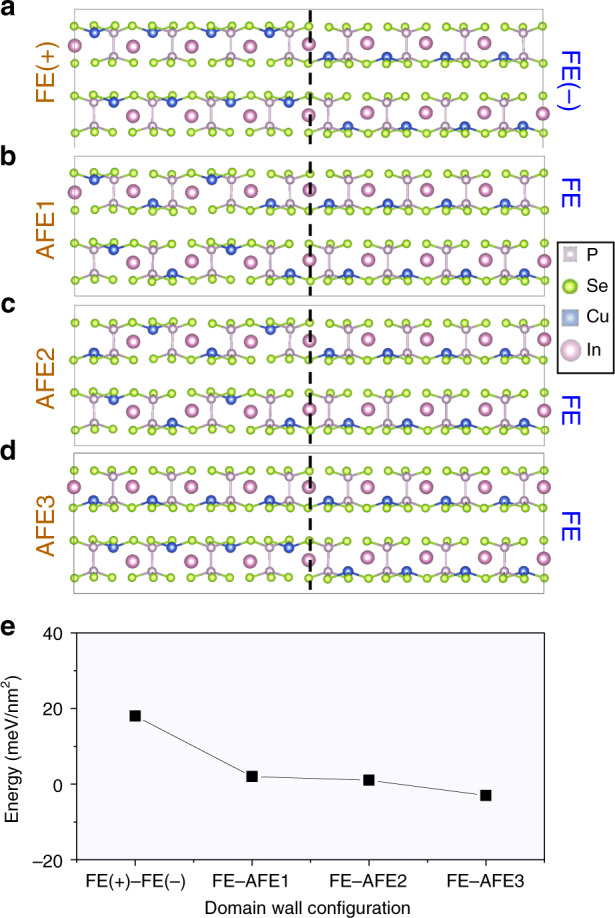
Calculated structures of domain walls and phase boundaries in CuInP_2_Se_6_. **a** Ferrielectric domain wall (FE/FE). **b** Three kinds of antiferroelectric/ferroelectric boundaries, respectively AFE1/FE (**b**), AFE2/FE (**c**) and AFE3/FE (**d**). **e** Corresponding boundary and wall energies. FE stands for ferrielectric and AFE for antiferroelectric order.

Further insight into the structure and energetics of the AFE and FE phases and the AFE/FE domain walls has been gained using DFT calculations of large supercells, which accommodate such boundaries. The energetics and structures of several possible AFE configurations are shown in Supplementary Fig. 4. Consistent with prior work^12^, under zero strain, all bulk AFE structures have a higher energy than the bulk FE structure. Meanwhile, calculated energies of all three types of AFE/FE boundaries are small, within 2 meV/nm^2^ of each other, while the energy of a FE/FE domain wall is much higher, 21 meV/nm^2^ (Fig. 5). This result indicates that the formation of the observed mixed AFE/FE state is largerly  governed by the energetics of the respective domains and that there is a high likelihood of formation of AFE/FE boundaries.

## Discussion

The complete analysis of phase coexistence CuInP_2_Se_6 _falls outside the scope of this manuscript. However, we have carried out analytical modeling of the mixed free energy functional within the Landau–Ginzburg–Devonshire framework (Supplementary Discussion). The functional incorporates the contributions to the free energy from ferroelectric, antiferroelectric, antiferrodistortive order parameters, as well as coupling and striction terms. Importantly, although the coexistence of antiferroelectric and ferroelectric order parameters is intuitively simple, its detailed functional is very complicated. The variation of the order parameter across the domain wall is opposite for the ferroelectric and antiferroelectric end-states: in ferroelectrics, polarization drops to zero at the domain wall (*x* = *x*_0_ in Supplementary Eq. (18)), while the antiferroelectric order parameter A increases at the domain wall (*x* = *y*_0_ in Supplementary Eq. (19)). Our experimental observable is strain, and specifically piezoresponse measured as a voltage derivative of strain. In the case of the FE/AFE boundary we do indeed predict a maximum strain at the wall within the approximations of the model (Supplementary Fig. 7). This behavior can be rationalized by analogy with double-hysteresis strain loops in macroscopic antiferroelectric switching (schematic in Fig. 3d). Upon onset of polarization (transition from AFE to FE phase), the lattice experiences maximum deformation (expansion in perovskite oxides^29,30^) that exceeds subsequent deformation in the ferroelectric state (due to piezoresponse). In our interpretation, the AFE–FE domain boundary in CuInP_2_Se_6_ is precisely the region of phase separation. The result of the LGD modeling, showing maximum strain at the boundary, supports the analogy between AFE/FE transition due to electric field (in macroscopic double-hysteresis loops) and due to phase separation in real-space (in our measurements). Although we cannot yet model the piezoresponse (dynamic strain), it is also maximized in the macroscopic hysteresis loops at the AFE/FE switching, and could therefore be maximum in our measurements in Fig. 1 and 2. Based on these results, piezoelectric domain walls should also be generally found in partially polarized antiferroelectrics.

Using quantitative piezoelectric microscopy, combined with DFT calculations and optical spectroscopy, we have revealed unusual domain-wall properties in polar CuInP_2_Se_6_, where domain walls exhibit maximum piezoresponse at the domain walls in contrast to the expectations for ferroelectrics. Whereas polar walls were previously detected in in antiferroelectric PbZrO_3_^31^ and in ferroelastic CaTiO_3_^32,33^, we have inferred that in CuInP_2_Se_6_ the domain walls separate regions of antiferroelectric and ferrielectic ordering. These domain walls should be general for antiferroelectrics while being distinct from domain walls in either ferroelectric or antiferroelectric phases, thus emerging as a new entity in the context of domain-wall electronics^19^. Meanwhile, the observation of antiferroelectric state confirms theoretical modeling of very small energy differences between ferrielectric and antiferroelectic states in CuInP_2_Se_6_. The ability to define and control locally polarized, mesoscale regions in an otherwise non-polar matrix may provide a path to integrate ferroic and electronic functionality via van der Waals interfaces, which is complementary to ferroelectric materials and that may persist down to single layer^12^.

## Methods

### Crystal growth

The single crystal CuInP_2_Se_6_ was grown from gas phase by chemical transport reactions. Iodine has been used as transport agent with concentration of 4–5 mg/cm^3^. The temperature of evaporation zone was 870 K, and for the crystallization zone temperature equals 850 K. The duration of the growth process was 350 h. The resulting product was thin single crystal plates with dimensions near 10 × 5 × 0.1 mm^3^. Cu_0.4_In_1.2_P_2_S_6_ single crystals were synthesized through the vapor transport method. Starting materials, sealed in fused silica ampules, were heated to 750–775 °C at a rate of 30 °C/h and held at that temperature for 4 days and then cooled at a rate of 20 °C/h.

### PFM imaging

Ultrahigh vacuum contact PFM imaging and polarization switching were performed on an Omicron AFM/STM, interfaced with a Nanonis controller package. The chamber pressure was 1 × 10^−10^ mbar or better. The samples were mounted on a standard Omicron sample plate and affixed with a silver conductive epoxy (Epo-Tek EJ2189-LV). A clean surface was prepared by Scotch tape method in UHV. Images of CuInP_2_S_6_ shown in this paper were acquired in an Ar-filled glove-box using Bruker-Icon AFM. The freshly cleaved surface was likewise prepared shortly before the measurement.

### Single crystal XRD

The structure of single crystals was characterized by using a Rigaku XtalLAB PRO diffractometer with graphite monochromatized Mok-alpha radiation (lambda = 0.71073 Angstrom) equipped with a Dectris Pilatus 200 K and an Oxford Cryosystems N-HeliX cryocooler for temperature ranging from 100 to 293 K.

### Raman spectroscopy

The Raman spectra were measured in a custom-built micro-Raman setup. The samples were excited with a continuous wave (cw) diode-pumped solid-state laser (Excelsior, Spectra Physics, 532 nm, 100 mW) through an upright microscope using a ×50 long-working distance objective with NA (numeric aperture) = 0.5. The typical incident laser power on a sample was maintained at ~ 100 μW to reduce possible laser heating and damaging of the samples during Raman spectra acquisition. The scattered Raman light was analyzed by a spectrometer (Spectra Pro 2300i, Acton, *f* = 0.3 m) that was coupled to the microscope and equipped with a 1800 groves/mm grating and a CCD camera (Pixis 256BR, Princeton Instruments). The low-temperature Raman spectra were measured using a liquid He-cryostat (MicrostatHiResII, Oxford Instruments) with a temperature controller (MercuryiT, Oxford Instruments) that allowed precise control from 3.6 to 300 K. Raman spectra were sampled with 10 K step from 100 to 250 K, and also at 4, 7, and 20 K. The cryostat was mounted on a motorized *XY* microscope stage (Marzhauser) under the microscope of the micro-Raman setup. The cryostat was evacuated to the base pressure of 7 × 10^−7^ mbar prior to cool down.

### Second harmonic measurements

Second harmonic generation (SHG) measurements were conducted using a 50 fs Ti:sapphire laser (Micra, Coherent) at 800 nm and 80 MHz repetition rate. The laser beam was passed through a half-wave plate mounted in a rotation stage and was directed into an upright microscope (Olympus) and focused onto a sample surface using a ×50 microscope objective (Numerical Aperture: NA = 0.5) to a few micron spot. The laser energy at the sample surface was ~0.1 W. The SHG light was collected in backscattering configuration using the same objective and was directed to a monochromator (Spectra Pro 2300i, Acton, *f* = 0.3 m) that was coupled to the microscope and equipped with a 150 grooves/mm grating and a CCD camera (Pixis 256BR, Princeton Instruments). Before entering the monochromator, the SHG light was passed through a short-pass cutoff filter (650 nm) and a polarizer to filter out the fundamental excitation light at 800 nm and select the SHG polarization parallel to that of the excitation light. The low-temperature SHG measurements were conducted using the same liquid He-cryostat, which was used for Raman measurements.

### DFT calculations

The DFT calculations (relaxations and Γ-point phonon frequencies) in this study use the VASP 5.3.5 computational package^34^ and are carried out under the Perdew–Burke–Ehrenhof generalized gradient approximation (GGA) and the the DFT-D2 method as developed by Grimme^35^. The recommended VASP PAW pseudopotentials were used. All calculations used a 600 eV energy cutoff. Raman frequencies and intensities are calculated using the package developed by Fonari and Stauffer^36^. The calculations were performed on two phases of CuInP_2_Se_6_: a 20-atom ferroelectric (FE) phase, and a 40-atom 1 × 2 × 1 antiferrolectric (AFE) phase. The setup for the Cu atoms in the AFE unit cell is the same as that found by Song et al.^12^ For phonon calculations, both structures were relaxed so that the forces are less than 5e^−8^ eV/Å to eliminate residual spurious forces. The FE/FE and FE/AFE domain-wall calculations are performed using 8 × 2 × 1 supercells containing 160 atoms. These calculations use a Γ-centered Monkhorst-Pack (MP) k-point grid of 1 × 4 × 2. All atoms were relaxed until all forces were smaller than 0.02 eV/Å. The domain boundary energy per unit area is calculated as

1 $$E_{{\rm{boundary}}} = (E_{{\rm{total}}} - E_{{\rm{phase}}_2})/2S,$$

where *E*_total_ is the supercell energy, *E*_phase1_ and *E*_phase2_ are the energies for the requisite number of atoms in those phases, and *S* is the cross-sectional area of the boundary.

## Supplementary information

Supplementary Information

## Data Availability

Source data for the scanning probe images, results of optical spectroscopy and first principles calculations presented in this manuscript will be shared upon reasonable request.

## Code availability

The data analysis was carried out with widely accepted commercial and open source software without any significant code customization.
