# Supplementary material for: Piezoelectric domain walls in van der Waals antiferroelectric CuInP2Se6
Source: Nat Commun. 2020 Jul 17;11:3623. doi: 10.1038/s41467-020-17137-0 (PMC7368031; doi:10.1038/s41467-020-17137-0)
Supplement: Supplementary file 1 — Supplementary Information [file 41467_2020_17137_MOESM1_ESM.docx]

Supplementary Information

**Piezoelectric domain walls in van der Waals antiferroelectric CuInP_2_Se_6_**

*Andrius Dziaugys^1*^, Kyle Kelley^2*^, John A. Brehm^3^, Lei Tao^3,4^, Alexander Puretzky^2^, Tianli Feng^2,3^, Andrew O’Hara,^3^ Sabine Neumayer^2^, Marius Chyasnavichyus^2^, Eugene A. Eliseev^5^, Juras Banys^1^, Yulian Vysochanskii^6^, Feng Ye^7^, Bryan C. Chakoumakos^7^, Michael A. Susner^8,9^, Michael A. McGuire^10^, Sergei V. Kalinin^2^, Panchapakesan Ganesh^2^, Nina Balke^2^, Sokrates T. Pantelides^3,11^, Anna N. Morozovska^12^ and Petro Maksymovych^2†^*

^1^Faculty of Physics, Vilnius University, Vilnius, LT-01513 Lithuania

^2^The Center for Nanophase Materials Sciences, Oak Ridge National Laboratory, Oak Ridge, TN, 37831, USA

^3^ Department of Physics and Astronomy and Department of Electrical Engineering and Computer Science, Vanderbilt University, Nashville, Tennessee 37235, USA

^4^University of Chinese Academy of Sciences & Institute of Physics, Chinese Academy of Sciences, Beijing, China.

^5^Institute for Problems of Materials Science, National Academy of Sciences of Ukraine, Krjijanovskogo 3, 03142 Kyiv, Ukraine

^6^Institute of Solid State Physics and Chemistry, Uzhgorod University, 88000 Uzhgorod, Ukraine

^7^Neutron Scattering Division, Oak Ridge National Laboratory, Oak Ridge, TN, USA

^8^Materials and Manufacturing Directorate, Air Force Research Laboratory, WPAFB, Ohio 45433

^9^UES, Inc. 4401 Dayton-Xenia Rd., Dayton, OH 45432

^10^Materials Science and Technology Division, Oak Ridge National Laboratory, Oak Ridge, Tennessee 37831, USA

^11^Department of Electrical Engineering and Computer Science, Vanderbilt University, Nashville, Tennessee 37235, USA

^12^Institute of Physics, National Academy of Sciences of Ukraine, Prospect Nauky 46, Kyiv-28, 03680, Ukraine

*^†^corresponding author. Email: maksymovychp@ornl.gov*

**equal contribution*

**Supplementary Figures:**


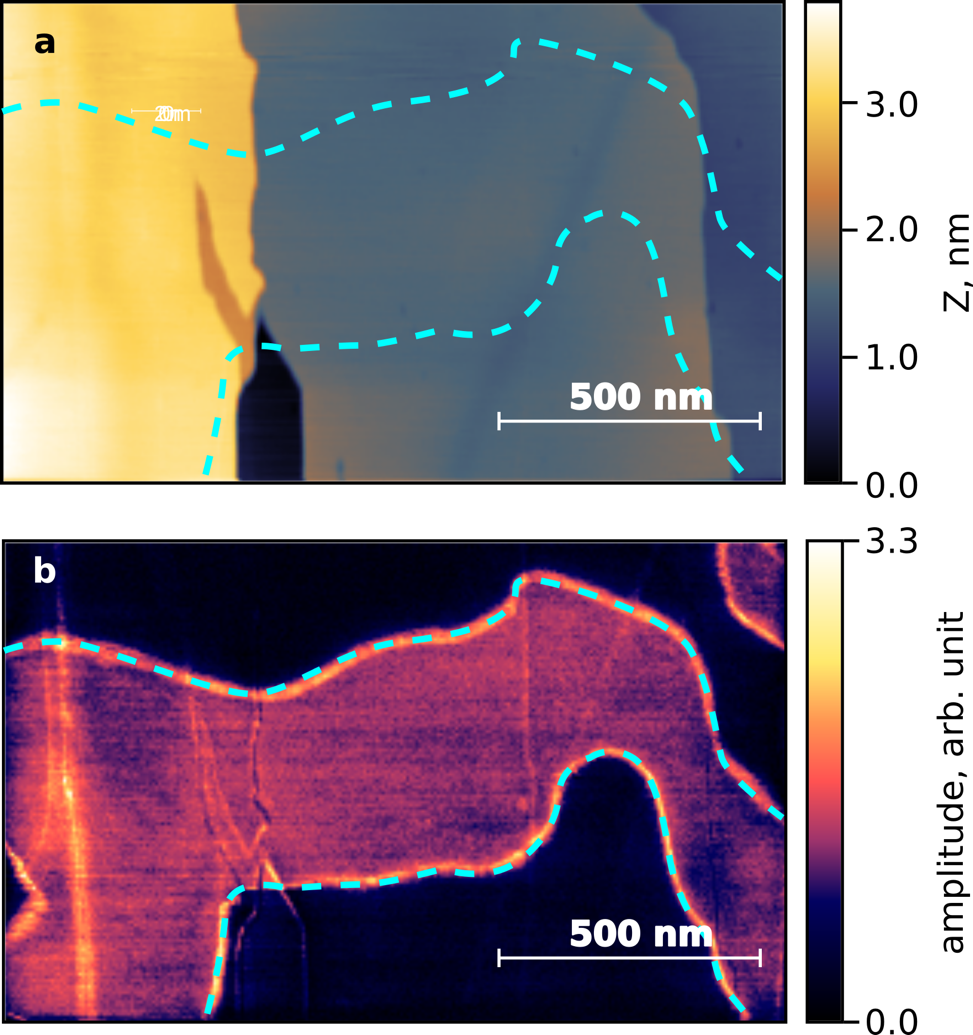


**Supplementary Figure 1**. Minimal effects of topographic cross-talk on piezoelectric signal of domain walls. Topography (a) and piezoresponse amplitude (b) of a freshly cleaved CuInP_2_Se_6_ crystal surface in ultrahigh vacuum. Very little correlation between topography and piezoresponse is observed.

**Supplementary Figure 2.** Region of CuInP_2_Se_6_ surface characterized by contact Kelvin force microscopy. (a) Band excitation piezoresponse image of freshly cleaved CuInP_2_Se_6_ surface at 140 K in ultrahigh vacuum with inset of spectroscopy grid location. (b) 30x30 contact Kelvin force spectroscopy grid displaying data slice at -2V. (c) K-means clustering of contact Kelvin force microscopy used to derive Figure 3(c).


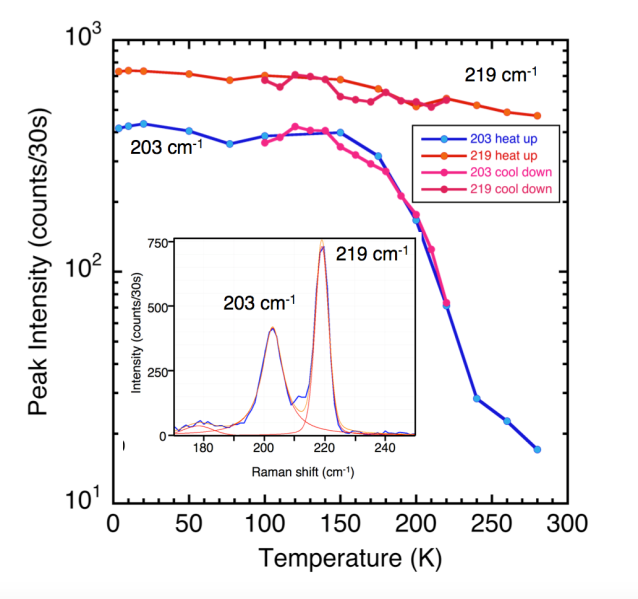


**Supplementary Figure 3.** Tracking Raman peaks at 219 cm^-1^ and 203 cm^-1^ across the phase transition in CuInP_2_Se_6_.


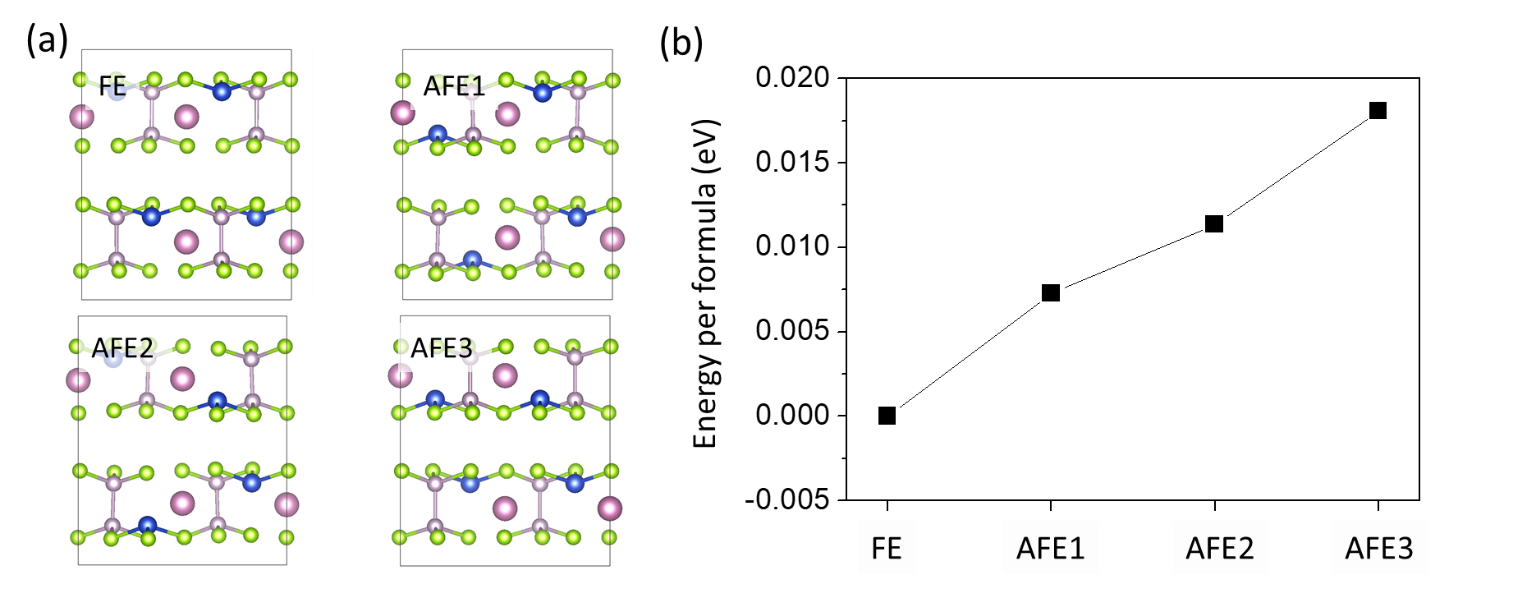


**Supplementary Figure 4.** First principles modeling of possible ordered structures of CuInP_2_Se_6_. (a) Atomic structures of ferrielectric state (FE) and three types of anti-ferroelectric state (AFE1, AFE2 and AFE3) in the bulk. (b) Energy per formula of AFE1, AFE2 and AFE3 relative to the ferroelectric ground state FE.

**Supplementary Figure 5.** Layered structures of CuInP_2_Se_6_ inferred from X-ray diffraction. Structures at (a) 100 K and (b) 293 K. Blue atoms are Cu, pink atoms are In, yellow atoms are Se, and grey atoms are P. (c) View ([001] direction) of a single CuInP_2_Se_6_ layer showing the arrangement of Cu (blue), In (pink), P (grey) and Se (yellow). Atomic displacement of individual atoms of CuInP_2_Se_6_ at (d) 100 K, (e) 180 K and (f) 250K

**Supplementary Figure 6. Analysis of atomic displacements across phase transition in CuInP_2_Se_6_.** Temperature dependence of anisotropic atomic displacement parameters of Cu, In, P and Se in CuInP_2_Se_6_ a) and c); Temperature dependence of Cu ordering in the middle of an octahedron (high temperature) and near the edge (low temperature); d) Cu and In positions with respect to the octahedral center.


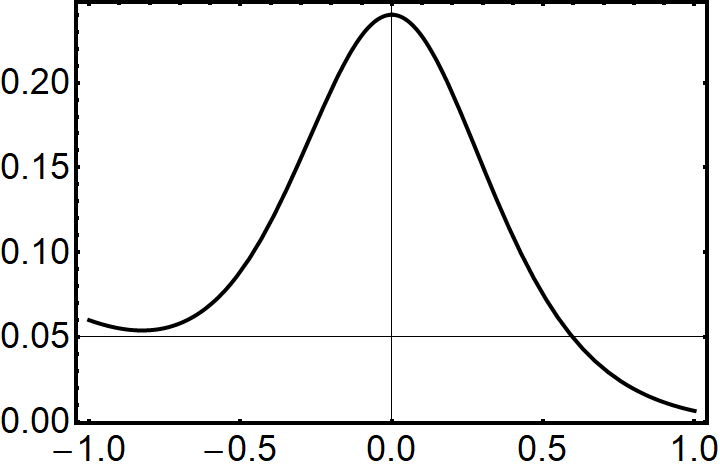


Coordinate *x*/*L_c_*

Local strain (arb. units)

0)

AFE-phase

FE-phase

**Supplementary Figure 7.** Local strain across AFE-FE boundary at x=0 from analytical modeling.

**Supplementary Tables:**

**Supplementary Table 1**. a) Structural parameters of the single crystal CuInP_2_Se_6_ in *P*31*c* (No. 159) phase at 100K. Total of 145603 reflections were collected. b) Structural parameters of the single crystal CuInP_2_Se_6_ in *P*31*c* (No. 159) phase at 180K. Total of 14831 reflections were collected c) Structural parameters of the single crystal CuInP_2_Se_6_ in $P\bar{3}1c$ (No. 163) phase at 250K. A total of 11996 reflections were collected.

| (a)  T = 100 K | a = 6.402(2) Å, c = 13.319(6) Å, V = 472.8(5) Å^3^ . The agreement factor R_1_ = 2.66% was achieved by using 708 unique reflections with I > 4σ and resolution of d_min_ = 0.65 Å. Anisotropic atomic displacement parameters were used for all elements. | | | | | |
| --- | --- | --- | --- | --- | --- | --- |
|  | Site | *x* | *y* | *z* | Occupancy | *U_eq_*(Å^2^) |
| Cu | 2*b* | 2/3 | 1/3 | 0.1474(2) | 1 | 0.0173(4) |
| In | 2*a* | 0 | 0 | 0.2591(1) | 1 | 0.0087(2) |
| P1 | 2*b* | 1/3 | 2/3 | 0.3256(3) | 1 | 0.0057(4) |
| P2 | 2*b* | 1/3 | 2/3 | 0.1579(2) | 1 | 0.0057(4) |
| Se1 | 6*c* | 0.3013(1) | 0.3228(1) | 0.1096(1) | 1 | 0.0069(1) |
| Se2 | 6*c* | 0.3552(1) | 0.0054(1) | 0.3706(1) | 1 | 0.0077(2) |

| (b)  T = 180 K | a = 6.410(8) Å, c = 13.337(20) Å, V = 474.6(1.3) Å^3^ . The agreement factor R_1_ = 4.58% was achieved by using 706 unique reflections with I > 4σ and resolution of d_min_ = 0.65 Å. Anisotropic atomic displacement parameters were used for all elements. | | | | | |
| --- | --- | --- | --- | --- | --- | --- |
|  | Site | *x* | *y* | *z* | Occupancy | *U_eq_*(Å^2^) |
| Cu | 2*b* | 2/3 | 1/3 | 0.1526(4) | 1 | 0.045(1) |
| In | 2*a* | 0 | 0 | 0.2571(2) | 1 | 0.0136(3) |
| P1 | 2*b* | 1/3 | 2/3 | 0.3248(4) | 1 | 0.0082(9) |
| P2 | 2*b* | 1/3 | 2/3 | 0.1587(4) | 1 | 0.0085(9) |
| Se1 | 6*c* | 0.3059(2) | 0.3248(2) | 0.1097(1) | 1 | 0.0119(3) |
| Se2 | 6*c* | 0.3531(2) | 0.0046(2) | 0.3706(1) | 1 | 0.0128(3) |

| (c)  T = 250 K | a = 6.397(1) Å, c = 13.340(5) Å, V = 472.8(3) Å^3^ . The agreement factor R_1_ = 3.80% was achieved by using 538 unique reflections with I > 4σ and resolution of d_min_ = 0.65 Å. Anisotropic atomic displacement parameters were used for all elements. | | | | | |
| --- | --- | --- | --- | --- | --- | --- |
|  | Site | *x* | *y* | *z* | Occupancy | *U_eq_*(Å^2^) |
| Cu1 | 2*d* | 2/3 | 1/3 | 1/4 | 0.354(6) | 0.063(3) |
| Cu2 | 4*f* | 2/3 | 1/3 | 0.3334(6) | 2x0.323(6) | 0.063(3) |
| In | 2*a* | 0 | 0 | 1/4 | 1 | 0.0217(3) |
| P | 4*f* | 1/3 | 2/3 | 0.1662(1) | 1 | 0.0133(3) |
| Se | 12*i* | 0.33217(8) | 0.33730(7) | 0.12006(4) | 1 | 0.0211(2) |

**Supplementary Characterization:**

CuInP_2_Se_6_ has the lamellar structure^1^, characteristic of layered thiophosphates^2^. Full data sets were collected in temperature range from 100 K to 300 K and the structures were refined using SHELX-97 software (**Supplementary Table 1**). The bulk phase of CuInP_2_Se_6_ belong to the non-centrosymmetric space group *P*31*c* (No. 159) at 100 K and 180 K (**Supplementary Table 1a**)). At 100 K, about 93% of the Cu^+^ ions are in a well-defined off-centre position, displaced by 1.38 Å along the c axis (**Supplementary Figure 5d**)). The remaining 7% of Cu^+^ ions are still disordered within the layer as before. In^3+^ shifts in opposite direction when the temperature is lowered below T_c_, by about 0.14 Å at 100 K (**Supplementary Figure 5d**)). At 250K, the best fit is to centrosymmetric space group *P*$\bar{3}$1*c* (No. 163) (**Supplementary Table 1c**)). These results largely agree with previous work^3,4^. At room temperature, 43% of Cu^+^ ions occupy the central position and another 57% are found near the edges of the cage. This indicates order-disorder type ferroelectric ordering in CuInP_2_Se_6_. The phase transition is also directly detected by Raman and SHG spectroscopies, carried out on freshly cleaved crystalline flakes of CuInP_2_Se_6_. As seen in **Figure 4** and **Supplementary Figure 3**, Raman spectroscopy clearly detects the phase transition below ~250K. The peaks at 203 and 219 cm^-1^ appear first at around 250K and their integrated intensity continues to grow, eventually saturating below ~180K (**Supplementary Figure 3**). Simultaneously measured SHG intensity (**Figure 4d**) shows a rapid increase below 220K.

**Supplementary Discussion:**

**Free energy and equations of state**

In the classical Ginzburg-Landau-Devonshire (LGD) approach, two vectorial long-range order parameters, polarization components $P_{i}$ and structural order (SO) parameter $\Phi_{i}$, will be used for the description of the (anti)ferrodistortive (AFD), ferroelectric (FE), and antiferroelectric (AFE) long-range orders in a multiferroic. The bulk part of LGD thermodynamic potential consists of the following contributions:

$G=\int d^{3}x\left( \Delta G_{AFD}+\Delta G_{FE}+\Delta G_{BQC}+\Delta G_{ST}+\Delta G_{EL} \right)$. (1)

The compact form of the AFD contribution is^5^:

$\Delta G_{AFD}=b_{i}\Phi_{i}^{2}+b_{ij}\Phi_{i}^{2}\Phi_{j}^{2}+b_{ijk}\Phi_{i}^{2}\Phi_{j}^{2}\Phi_{k}^{2}+v_{ijkl}\frac{\partial\Phi_{i}}{\partial x_{k}}\frac{\partial\Phi_{j}}{\partial x_{l}}$ . (2)

We assume that the coefficients $b_{i}$ can be temperature dependent, but the AFD transition temperature $T_{\Phi}$ is significantly higher than the room temperature of the observations.

The compact form of the FE and AFE contributions are:

$\Delta G_{FE}=a_{i}\left( P_{i}^{2}+A_{i}^{2} \right)+a_{ij}\left( P_{i}^{2}P_{j}^{2}+A_{i}^{2}A_{j}^{2} \right)+a_{ijk}\left( P_{i}^{2}P_{j}^{2}P_{k}^{2}+A_{i}^{2}A_{j}^{2}A_{k}^{2} \right)+\gamma_{ij}^{ab}\left( P_{i}P_{j}-A_{i}A_{j} \right)+g_{ijkl}^{aa}\left( \frac{\partial P_{i}}{\partial x_{k}}\frac{\partial P_{j}}{\partial x_{l}}+\frac{\partial A_{i}}{\partial x_{k}}\frac{\partial A_{j}}{\partial x_{l}} \right)+g_{ijkl}^{ab}\left( \frac{\partial P_{i}}{\partial x_{k}}\frac{\partial P_{j}}{\partial x_{l}}-\frac{\partial A_{i}}{\partial x_{k}}\frac{\partial A_{j}}{\partial x_{l}} \right)$, (3)

where the FE and AFE order parameters, $P_{i}=\frac{1}{2}\left( P_{i}^{a}+P_{i}^{b} \right)$ and $A_{i}=\frac{1}{2}\left( P_{i}^{a}-P_{i}^{b} \right)$, are introduced, $P_{i}^{a}$ and $P_{i}^{b}$ are the polarization components of two equivalent sublattices “*a*” and “*b*”.^6^ As usual for proper and incipient ferroelectrics, the coefficients $a_{k}$ are temperature dependent and obeys the linear law, $a_{k}\left( T \right)=\alpha_{T}\left[ T-T_{C} \right]$, where $T_{C}$ is the Curie temperature, and *T* is the absolute temperature; at that negative $a_{k}\left( T \right)$ supports FE or AFE state. The sign and value of $\gamma_{ij}^{ab}$ determines the AFE and FE phases coexistence.

The compact form of the biquadratic coupling energy between polarization and SO is

$\Delta G_{BQC}=\xi_{ijkl}^{aa}\left( P_{i}P_{j}+A_{i}A_{j} \right)\Phi_{k}\Phi_{l}+\xi_{ijkl}^{ab}\left( P_{i}P_{j}-A_{i}A_{j} \right)\Phi_{k}\Phi_{l}$, (4)

where poorly known tensorial biquadratic coupling coefficients, $\xi_{ijkl}^{aa}$ and $\xi_{ijkl}^{ab}$, are usually treated as fitting parameters to experiment.

Electrostriction and rotostriction contributions are

$\Delta G_{ST}=-Q_{ijkl}^{aa}\sigma_{ij}\left( P_{k}P_{l}+A_{k}A_{l} \right)-Q_{ijkl}^{ab}\sigma_{ij}\left( P_{k}P_{l}{-A}_{k}A_{l} \right)-R_{ijkl}\sigma_{ij}\Phi_{k}\Phi_{l}$, (5)

where $\sigma_{ij}$ are elastic stress tensor components, which satisfy the equation of mechanical equilibrium, $\frac{\partial\sigma_{ij}}{\partial x_{j}}=0$. Electrostriction and rotostriction coefficients are, $Q_{ijkl}^{aa}$, $Q_{ijkl}^{ab}$ and $R_{ijkl}$, respectively. Elastic and flexoelectric contributions are

$\Delta G_{EL}=-\frac{1}{2}s_{ijkl}\sigma_{ij}\sigma_{kl}-{\frac{1}{2}F}_{ijkl}\left( \sigma_{ij}\frac{\partial P_{k}}{\partial x_{l}}-P_{k}\frac{\partial\sigma_{ij}}{\partial x_{l}} \right)$ (6)

Here $s_{ijkl}$ are the components of elastic compliances tensor (see e.g. Supplementary Reference [7]); $F_{ijkl}$ are flexoelectric tensor components.

For a general case of inhomogeneous (e.g. domain structured or/and spatially modulated) system, one should solve the coupled Euler-Lagrange equations of states, which are expressed via the variational derivatives of the functional (**Supplementary Equation 1**):

$\frac{\delta G}{\delta\Phi_{i}}=0$, $\frac{\delta G}{\delta P_{i}}=-E_{i}$, $\frac{\delta G}{\delta A_{i}}=0$. (7)

which solution is equivalent to the minimization of the free energy functional (**Supplementary Equation 1**), that defines the distribution of the order parameter fields given the boundary conditions on the external surfaces. External $E_{i}^{ext}$ and depolarization fields $E_{i}^{d}$, which contribute to the electric field, $E_{i}=E_{i}^{ext}$+$E_{i}^{d}$, can be found from electrostatic equations. Elastic fields, which are, in fact, the secondary order parameters, satisfy equation of state and mechanical equilibrium equations:

$\frac{\partial G}{\partial\sigma_{ij}}=-u_{ij}$, $\frac{\partial\sigma_{ij}}{\partial x_{j}}=0$ . (8)

The strain (or stress) should be defined at the system boundaries. Note that $P_{i}$ and $A_{i}$ can be strongly affected by the composition fluctuations, local elastic fields $\sigma_{ij}$ and $u_{ij}$ via the rotostriction and electrostriction couplings, as well as by depolarization field $E_{i}^{d}$.

Coupled equations (**Supplementary Equation 7, 8**), in their explicit form, give us the relations between the order parameters:

$2\left( b_{i}+b_{ij}\Phi_{j}^{2}+b_{ijk}\Phi_{j}^{2}\Phi_{k}^{2} \right)\Phi_{i}+\left( {\xi_{mjki}^{P}P}_{m}P_{j}+\xi_{mjki}^{A}A_{m}A_{j}-R_{mjki}\sigma_{mj} \right)\Phi_{k}-v_{ijkl}\frac{\partial^{2}\Phi_{j}}{\partial x_{k}\partial x_{l}}=0$, (9)

where $\xi_{mjkl}^{P}=\xi_{mjkl}^{aa}+\xi_{mjkl}^{ab}$ and $\xi_{mjkl}^{A}=\xi_{mjkl}^{aa}-\xi_{mjkl}^{ab}$, and no summation on “*i*”.

$2\left( a_{i}+a_{ij}P_{j}^{2}+a_{ijk}P_{j}^{2}P_{k}^{2} \right)P_{i}+\left( \gamma_{ik}^{ab}-Q_{mjki}^{P}\sigma_{mj}{+\xi}_{ikml}^{P}\Phi_{m}\Phi_{l} \right)P_{k}-g_{ijkl}^{P}\frac{\partial^{2}P_{j}}{\partial x_{k}\partial x_{l}}-F_{mjil}\frac{\partial\sigma_{mj}}{\partial x_{l}}=E_{i}$, (10)

where $g_{ijkl}^{P}=g_{ijkl}^{aa}+g_{ijkl}^{ab}$ and $Q_{ijkl}^{P}=Q_{ijkl}^{aa}+Q_{ijkl}^{ab}$, and no summation on “*i*”.

$2\left( a_{i}+a_{ij}A_{j}^{2}+a_{ijk}A_{j}^{2}A_{k}^{2} \right)A_{i}+\left( {-\gamma}_{ik}^{ab}-Q_{mjki}^{A}\sigma_{mj}{+\xi}_{ikml}^{A}\Phi_{m}\Phi_{l} \right)A_{k}-g_{ijkl}^{A}\frac{\partial^{2}A_{j}}{\partial x_{k}\partial x_{l}}=0$, (11)

where $g_{ijkl}^{A}=g_{ijkl}^{aa}-g_{ijkl}^{ab}$ and $Q_{ijkl}^{A}=Q_{ijkl}^{aa}-Q_{ijkl}^{ab}$, and no summation on “*i*”.

Local strain is the following

${u_{ij}=s}_{ijkl}\sigma_{kl}+F_{ijkl}\frac{\partial P_{k}}{\partial x_{l}}-V_{ij}N_{d}+Q_{ijkl}^{P}P_{k}P_{l}+Q_{ijkl}^{A}A_{k}A_{l}+R_{ijkl}\Phi_{k}\Phi_{l}$ . (12)

**Appendix A.2: FE and AFE domain structures**

Since “aa” constants are for next-nearest neighbors sublattices, while “ab” constants are for the nearest neighbors sublattices, one can assume that, $\left| g_{ijkl}^{aa} \right|\ll\left| g_{ijkl}^{ab} \right|$. As a rule, the biquadratic coupling to the polar subsystem is small, and depolarization field is absent for uncharged walls, we obtained decoupled system of equations:

$2\left( a_{i}+a_{ij}P_{j}^{2}+a_{ijk}P_{j}^{2}P_{k}^{2} \right)P_{i}+\gamma_{ik}^{ab}P_{k}-g_{ijkl}^{ab}\frac{\partial^{2}P_{j}}{\partial x_{k}\partial x_{l}}\approx0$, (13)

$2\left( a_{i}+a_{ij}A_{j}^{2}+a_{ijk}A_{j}^{2}A_{k}^{2} \right)A_{i}{-\gamma}_{ik}^{ab}A_{k}+g_{ijkl}^{ab}\frac{\partial^{2}A_{j}}{\partial x_{k}\partial x_{l}}\approx0$, (14)

${\delta u}_{ij}\approx F_{ijkl}\frac{\partial P_{k}}{\partial x_{l}}-V_{ij}N_{d}+Q_{ijkl}^{aa}\left( P_{k}P_{l}-A_{k}A_{l} \right)$. (15)

For a 1D case of the second order ferroelectrics with $a_{ijk}=0$, we obtained from **Supplementary Equations 13-15**:

$\left( \alpha+\gamma\right)P+\beta P^{3}-g\frac{\partial^{2}P}{\partial x^{2}}\approx0$, (16)

$\left( \alpha-\gamma\right)A+\beta A^{3}+g\frac{\partial^{2}A}{\partial x^{2}}\approx0$, (17)

where $\alpha={2a}_{1}$, $\beta=a_{11}$ , $\gamma=\gamma_{11}^{ab}$ and ${g=g}_{44}^{ab}$. The FE and AFE order parameters are introduced as $P=\frac{1}{2}\left( P_{3}^{a}+P_{3}^{b} \right)$ and $A=\frac{1}{2}\left( P_{3}^{a}-P_{3}^{b} \right)$, in-plane coordinate $x_{1}=x$, $P_{3}^{a}$ and $P_{3}^{b}$ are the out-of-plane polarization components of two equivalent sublattices “*a*” and “*b*”. Let us assume the natural boundary conditions at the remote boundaries, $\left. \frac{\partial P}{\partial x} \right|_{S}=\left. \frac{\partial A}{\partial x} \right|_{S}=0$.

For the case when parameters $\alpha+\gamma<$0, $\beta>0$ and $g>0$, the FE phase is absolutely stable. Corresponding solution of **Supplementary Equation 13** is

$P(x)=P_{b}\sqrt{\frac{2m}{1+m}}sn \left( \left. \frac{x-x_{0}}{L_{c}\sqrt{1+m}} \right|m \right)$ , (18)

where $P_{b}=\sqrt{{-\left( \alpha+\gamma\right)}/\beta}$ , $L_{C}=\sqrt{-g/\left( \alpha+\gamma\right)}$, $x_{0}$ and $0\leq m\leq1$ are free parameters to satisfy the boundary conditions. A limiting case of a single domain wall corresponds to $m=1$, and corresponding kink-type solution is given by expression $P(x)=P_{b}\tanh\left( \frac{x-x_{0}}{2L_{c}} \right)$. Hence solution (**Supplementary Equation 18**) describes "dark" FE domain walls of Ising-type.

For the case $\alpha-\gamma>$0, $\beta>0$ and $g>0$ the AFE phase is absolutely. The solution of **Supplementary Equation 14** is

$A(x)=A_{b}\sqrt{\frac{2n}{1-2n}}cn \left( \left. \frac{x-y_{0}}{L_{c}\sqrt{1-2n}} \right|2n \right)$, (19)

where $A_{b}=\sqrt{\left( \alpha-\gamma\right)/\beta}$ and $L_{C}=\sqrt{g/\left( \alpha-\gamma\right)}$; $y_{0}$ and $0\leq n\leq0.5$ are free parameters to satisfy the boundary conditions. Solution (**Supplementary Equation 19**) describes "bright" AFE domain walls, which are not observable, because only the FE parameter P is directly observable, while A is not the one.

However, there are indirect ways. Namely. assuming that $\left| Q_{ijkl}^{aa} \right|\ll\left| Q_{ijkl}^{ab} \right|$, the value of the electrostriction-induced local strain in the FE is $\delta{u_{ij}^{FE}\approx Q}_{ijkl}^{ab}P_{k}P_{l}$ (it is zero at the Ising-type FE wall, where **P** is small) and it is $\delta{u_{ij}^{AFE}\approx-Q}_{ijkl}^{ab}A_{k}A_{l}$ in the AFE (it is also maximal at the walls, where **A** is maximal). However, the strain difference at AFE-FE boundary is:

$\delta{u_{ij}=\delta u_{ij}^{FE}-{\delta u}_{ij}^{AFE}\approx Q}_{ijkl}^{ab}\left( P_{k}P_{l}+A_{k}A_{l} \right)$ (20)

The effective strain across the “bright” FE-AFE boundary is schematically shown in **Supplementary Figure 7**.

**Supplementary References:**

Maisonneuve, V., Cajipe, V. B., Simon, A., Von Der Muhll, R. & Ravez, J. Ferrielectric ordering in lamellar CuInP_2_S_6_. *Phys. Rev. B* **56**, 10860–10868 (1997).

Susner, M. A., Chyasnavichyus, M., McGuire, M. A., Ganesh, P. & Maksymovych, P. Metal Thio‐ and Selenophosphates as Multifunctional van der Waals Layered Materials. *Adv. Mater.* **29**, 1602852 (2017).

Vysochanskii, Yu. M., Molnar, A. A., Gurzan, M. I., Cajipe, V. B. & Bourdon, X. Dielectric measurement study of lamellar CuInP_2_Se_6_: successive transitions towards a ferroelectric state via an incommensurate phase? *Solid State Commun.* **115**, 13–17 (2000).

Vysochanskii, Y. M., Molnar, A. A., Gurzan, M. I. & Cajipe, V. B. Phase transitions in CuInP_2_(Se_x_S_1−x_)_6_ layered crystals. *Ferroelectrics* **257**, 147–154 (2001).

E. A. Eliseev, A. N. Morozovska, C. T. Nelson, and S. V. Kalinin, Intrinsic structural instabilities of domain walls driven by gradient coupling: Meandering antiferrodistortive-ferroelectric domain walls in BiFeO_3_. *Phys. Rev. B* **99**, 014112 (2019).

C. Kittel, Theory of antiferroelectric crystals, *Phys. Rev.* **82**, 729 (1951).

1. J.X. Zhang, Y.L. Li, Y. Wang, Z.K. Liu, L.Q. Chen, Y.H. Chu, F. Zavaliche, and R. Ramesh, Effect of substrate-induced strains on the spontaneous polarization of epitaxial BiFeO_3_ thin films, *J. Appl. Phys.* **101**, 114105 (2007).
